# Are surface and deep learning approaches associated with study patterns and choices among medical students? A cross-sectional study

**DOI:** 10.1590/1516-3180.2018.0200060818

**Published:** 2018-10-22

**Authors:** Álvaro Henrique de Almeida Delgado, João Paulo Rodrigues Almeida, Larissa Souza Borowski Mendes, Isabella Noceli de Oliveira, Oscarina da Silva Ezequiel, Alessandra Lamas Granero Lucchetti, Giancarlo Lucchetti

**Affiliations:** I MD. Physician and Research Collaborator in the Department of Medical Education, School of Medicine, Universidade Federal de Juiz de Fora (UFJF), Juiz de Fora (MG), Brazil.; II Undergraduate Medical Student and Research Collaborator in the Department of Medical Education, School of Medicine, Universidade Federal de Juiz de Fora (UFJF), Juiz de Fora (MG), Brazil.; III Undergraduate Medical Student and Research Collaborator in the Department of Medical Education, School of Medicine, Universidade Federal de Juiz de Fora (UFJF), Juiz de Fora (MG), Brazil.; IV MD. Physician and Research Collaborator in the Department of Medical Education, School of Medicine, Universidade Federal de Juiz de Fora (UFJF), Juiz de Fora (MG), Brazil.; V MD, PhD. Associate Professor, Department of Medical Education, School of Medicine, Universidade Federal de Juiz de Fora (UFJF), Juiz de Fora (MG), Brazil.; VI MD, PhD. Associate Professor, Department of Medical Education, School of Medicine, Universidade Federal de Juiz de Fora (UFJF), Juiz de Fora (MG), Brazil.; VII MD, PhD. Associate Professor, Department of Medical Education, School of Medicine, Universidade Federal de Juiz de Fora (UFJF), Juiz de Fora (MG), Brazil.

**Keywords:** Learning, Students, medical, Sleep, Teaching, Education, medical

## Abstract

**BACKGROUND:**

Different approaches to learning can exert considerable influence on the teaching-learning process in medical education. This study aimed to investigate the association of surface and deep learning with study patterns, preferred type of assessment, practices of cheating and quality of sleep among medical students.

**DESIGN AND SETTING:**

Cross-sectional study on medical students enrolled in all six years of a medical school in Juiz de Fora, Brazil.

**METHODS:**

Questionnaires were applied to evaluate learning approaches (R-SPQ-2F), study patterns, sources and choices, and quality of sleep. Students’ learning approaches (deep or surface) were assessed in relation to their study patterns, study resources, quality of sleep and whether they cheated in tests.

**RESULTS:**

Among the 710 students included, 43% frequently studied on the night before an exam, 65% had used psychostimulants to study and more than 46% had cheated in an exam. Regarding quality of sleep, most students (53.4%) reported that their quality of sleep was poor, such that 45.3% slept for fewer than five hours before an exam. Those who studied just prior to an exam, used class summaries, preferred multiple-choice questions and cheated during the test had a more surface-learning approach. On the other hand, those who read books, preferred practical exams and slept better had a deeper approach.

**CONCLUSION:**

The type of learning approach was associated with study patterns and choices among medical students. Educators need to be attentive to the type of learning their students use and think of measures that impact teaching and assessment methods.

## INTRODUCTION

Students’ motivation, educational strategies, types of assessment and different learning approaches are factors that are considered fundamental to medical education. They have an important influence on the teaching-learning process.^1^^,^^2^^,^^3^ Biggs^4^ defined learning approaches as the combination of motivation and strategy that students use in the learning process, which can be “surface” or “deep”.

The deep approach comprises the capacity to correlate new and previous knowledge, study comprehensively in order to obtain the “entire picture” and search for meaning and implications for the acquired knowledge.^5^ Its strategy is based on personal commitment to the learning process and its motivation is intrinsic, in the search for self-fulfillment.^4^ This contrasts with the surface approach, which is the tendency to choose the quickest way to accomplish the task, in which students study the material in a linear manner, do not ask in-depth questions, do not show proper interest in the subject and rely on memory instead of comprehension.^5^ Students with surface approaches tend to achieve the minimum necessary to avoid failure at school.^6^

Previous studies have pointed out that the same students can have varying approaches, depending on the subject that they are studying, levels of apprehension and work overload.^7^^,^^8^ Understanding these learning approaches could have important repercussions on students’ academic life. Mattick et al.^9^ showed that students with a deep approach tend to be more organized, monitor their studies better and exert a greater effort to learn, such that their performance in tests is better. The same results were found by McManus et al.,^10^ who found that students with a deep approach had better outcomes in final exams.

Another important factor that is seldom studied within medical education, and which seems to have an important influence on students’ learning and even on students’ health, is study patterns. It has been shown that medical students usually have a very exhausting routine with long self-study hours,^11^ poor sleep quality,^12^ high caffeine consumption^13^ and failure to efficiently manage their study time.^14^ This dysfunctional study routine seems to be closely associated with learning approaches. However, despite frequent anecdotal descriptions within the educational context, few studies (and, to our knowledge, none in Brazil) have evaluated this relationship. Understanding this relationship could help educators to identify learning approaches in order to provide interventions and make curricular changes that might help students’ academic performance and health outcomes.

## OBJECTIVE

Thus, the objective of this study was to investigate the associations of surface and deep learning approaches with study patterns, preferred type of assessment method, practices of cheating and quality of sleep among medical students. Our hypothesis was that students whose approach was more towards surface learning would tend to use study methods that required less work (e.g. summaries or cheating), would have poorer quality of sleep (due to studying more frequently on the night before exams) and would choose assessment methods that were more superficial (multiple-choice tests rather than simulations or clinical assessments).

## METHOD

### Study design, ethics and participants

This cross-sectional study was conducted in 2016 and included students from all six years of medical school at the Federal University of Juiz de Fora (Universidade Federal de Juiz de Fora, UFJF), in Juiz de Fora (MG), Brazil. Students who were not in Brazil due to exchange programs, who were doing their clerkship in another city, who were not present when data was collected, or who did not wish to participate were excluded.

The project was approved by the Research Ethics Committee of the University Teaching Hospital of UFJF, under report no. 1.147.798/2015. All participating students signed a consent form.

### Instruments

The self-report questionnaire that was used took approximately 20 minutes to fill out and collected the following data:

- Sociodemographic data: age, ethnicity, gender, marital status and course level in which students were enrolled.
- Study patterns and study resources: for this study, six questions were developed, dealing with:
  1. the number of hours dedicated to studies each week;
  2. means used for routine study (books and study guides, class notes, professors’ slides and internet research);
  3. methods used to study for tests (schemes, summaries, books, audios and videos);
  4. how often the students waited until just before the test to do their studying (five options, ranging from “never” to “always”);
  5. whether students cheated (“yes” or “no”); and
  6. how often students used stimulants while studying (five options, ranging from “never” to “always”) and the type of stimulant used (students were asked to declare which stimulant(s) they used).
- Quality of sleep and its relationship with students’ routines: four questions relating to students’ quality of sleep and its relationship with their studying were used:
  1. quality of sleep (four options, ranging from “very poor” to “very good”);
  2. how often students had problems staying awake while driving or at social events (four options, ranging from “never” to “three or more times a week”);
  3. how often students had problems sleeping due to worrying about tests (four options, ranging from “never” to “three or more times a week”); and
  4. how many hours of sleep students had on the night before a test (three options: “fewer than five hours”, “five to eight hours” or “more than eight hours”).
- Revised two-factor version of the Study Process Questionnaire (R-SPQ-2F): developed by Biggs et al.^2^ and validated for use in Brazil by Costa.^15^ This questionnaire is made up of two 10-item scales (deep approach and surface approach) in a Likert format (“never” to “always or almost always”). Each scale has two subscales that each consists of five questions referring to motivation and five questions referring to strategy. Thus, four categories are formed: deep motivation (e.g. “I find that at times studying gives me a feeling of deep personal satisfaction”); deep strategy (e.g. “I find most new topics interesting and often spend extra time trying to obtain more information about them.”); surface motivation (e.g. “My aim is to pass the course while doing as little work as possible”); and surface strategy (e.g. “I find that the best way to pass examinations is to try to remember answers to likely questions”). The responses are coded as 1 = “never” to 5 = “always or almost always” and the results range from 10 to 50 points for each scale. The “deep approach” scale score is based on the sum of the deep strategy subscale (five questions) and the deep motivation subscale (five questions), and higher scores denote use of a deeper approach. The score for the “surface approach” scale is based on the sum of the surface strategy subscale (five questions) and the surface motivation subscale (five questions), and higher scores denote use of a more surface approach).

### Statistical analysis

A descriptive analysis was carried out on each variable, using frequency and percentage, or mean and standard deviation. An inferential analysis was then conducted. We firstly compared the means of students’ learning approaches (deep or surface), according to their study patterns, study resources, quality of sleep and whether they cheated in tests. For this, t tests for independent samples were used. We then correlated students’ learning approaches with their preferred assessment using the Spearman correlation test. All analyses were performed using the Statistical Package for the Social Sciences (SPSS), version 21 (SPSS Inc.). P < 0.05 was considered significant.

## RESULTS

Out of the total of 1,007 students officially registered in this medical school, 710 students were included (response rate 70.5%): 265 (37.3%) in the preclinical phase (1^st^ and 2^nd^ years), 233 (32.8%) in the clinical phase (3^rd^ and 4^th^ years) and 212 (29.9%) in the clerkship phase (5^th^ and 6^th^ years). The majority of the students were female (55.4%), single (98.0%) and white (66.9%), and their mean age was 22.11 years (standard deviation, SD: 3.11).

Table 1 shows the students’ study patterns, study resources, quality of sleep and cheating in tests. Most students (61.7%) were studying for six or more hours per week outside of class activities and the aides that they used were most frequently books (68.5%), followed by class notes (25.6%) and teachers’ slides (23.4%). Almost 43% frequently or always studied on the night before an exam and they used class summaries that they themselves or colleagues wrote (67.7%), books (35.8%) and study schemes (11.1%) to prepare for exams. A total of 46% of the students had cheated in an exam and 65% had used psychostimulants to study (40% frequently or always). Among the students who reported using stimulants, 73.5% of them used coffee, 17.0% caffeine pills, 16.6% energy drinks, 7.2% guarana seed powder, 3.6% methylphenidate and 4% other stimulants. Regarding the students’ quality of sleep, most (53.4%) reported that it was poor or very poor and 45.3% reported that they had fewer than five hours of sleep before an exam.

**Table 1.** Students’ study patterns, study resources, quality of sleep and cheating in tests

	n	%
Hours of study per week		
1 to 2	29	4.1
3 to 4	78	11.0
4 to 5	81	11.4
5 to 6	84	11.8
More than 6	438	61.7
Routine means of study		
Books and study guides	486	68.5
Classroom notes	182	25.6
Professors’ slides	166	23.4
Internet	154	21.7
Use of stimulants		
No	249	35.1
Yes, once	21	3.0
Yes, rarely	157	22.1
Yes, frequently	206	29.1
Yes, always	76	10.7
Waiting until just before exam to study		
Never	27	3.8
Rarely	88	12.4
Sometimes	282	39.8
Frequently	270	38.1
Always	41	5.8
Methods used to study for tests		
Schemes	79	11.1
Summaries	481	67.7
Books	254	35.8
Audios	60	8.5
Videos	62	8.7
Cheating		
Yes	326	46.6
No	373	53.4
Quality of sleep		
Very poor	84	11.9
Poor	293	41.5
Good	271	38.4
Very good	58	8.2
How often students had problems staying awake while driving or at a social event		
Never	176	25.0
Less than once a week	191	27.1
1 to 2 times a week	211	29.9
3 or more times a week	127	18.0
How often students had problems sleeping because they were worried about a test		
Never	382	54.3
Less than once a week	127	18.0
1 to 2 times a week	121	17.2
3 or more times a week	74	10.5
How many hours students were sleeping on the night before an exam	318	45.3
Fewer than 5 hours	356	50.7
Between 5 and 8 hours	28	4.0

Table 2 shows a comparison of the students’ learning approaches (deep or surface) according to their study patterns, study resources, quality of sleep and if they cheated on tests. We found that those who had a more superficial learning approach were those who studied on the night before an exam used professors’ slides, class audios and class summaries, and those who cheated in tests. On the other hand, those using or reading books and sleeping better learned more deeply.

**Table 2.** Students’ learning approaches (deep or surface) according to their study patterns, study resources, quality of sleep and cheating in tests

	Biggs Deep R-SPQ-2F Mean (SD)	P	Biggs Surface R-SPQ-2F Mean (SD)	P
Study resources				
Books				
Yes	29.98 (6.60)	0.009	21.50 (5.87)	< 0.001
No	28.58 (6.37)		24.23 (6.77)	
Class notes				
Yes	29.33 (6.23)	0.634	22.36 (6.63)	0.999
No	29.61 (6.67)		22.36 (6.18)	
Professors’ slides				
Yes	27.73 (6.45)	< 0.001	24.25 (6.77)	< 0.001
No	30.09 (6.50)		21.79 (6.03)	
Internet				
Yes	28.91 (6.59)	0.183	22.22 (6.70)	0.755
No	29.71 (6.54)		22.40 (6.18)	
Methods used to study for tests				
Self-study schemes				
Yes	30.02 (7.05)	0.493	22.35 (7.48)	0.981
No	29.48 (6.50)		22.36 (6.14)	
Summaries				
Yes	28.89 (6.45)	< 0.001	22.86 (6.40)	0.002
No	30.90 (6.58)		21.31 (5.94)	
Books				
Yes	30.82 (6.40)	< 0.001	20.62 (5.61)	< 0.001
No	28.81 (6.54)		23.34 (6.45)	
Audios				
Yes	29.24 (6.55)	0.716	24.18 (6.51)	0.020
No	29.56 (6.56)		22.19 (6.25)	
Videos				
Yes	30.29 (7.36)	0.347	21.09 (6.08)	0.100
No	29.46 (6.47)		22.48 (6.30)	
Study characteristics				
Hours studied per week				
1-5 hours	27.50 (6.28)	< 0.001	23.58 (6.64)	< 0.001
6 or more	30.79 (6.41)		21.60 (5.95)	
Waiting until night before to study				
Frequently/always	26.68 (6.15)	< 0.001	24.61 (6.44)	< 0.001
Never/rarely/sometimes	31.73 (5.96)		20.58 (5.59)	
Use of stimulants				
Frequently/always	29.58 (6.99)	0.865	22.73 (6.46)	0.208
Never/rarely/sometimes	29.49 (6.26)		22.12 (6.18))	
Sleep				
Hours slept on night before an exam				
Up to 5 hours	29.15 (6.81)	0.156	22.92 (6.73)	0.049
5 or more	29.86 (6.37)		21.96 (5.90)	
Quality of sleep				
Very good/good	29.95 (6.43)	0.128	21.90 (6.05)	0.064
Poor/very poor	29.19 (6.67)		22.79 (6.48)	
Difficulty staying awake while driving or at social events				
Once or more per week	28.96 (6.61)	0.025	22.82 (6.36)	0.071
Less than once	30.08 (6.50)		21.96 (6.22)	
How often does the student have problems sleeping due to worrying about tests?				
Once or more per week	29.35 (6.88)	0.618	23.09 (6.59)	0.061
Less than once	29.63 (6.45)		22.10 (6.15)	
Cheating				
Do you cheat?				
Yes	28.19 (6.25)	< 0.001	23.45 (6.33)	< 0.001
No	31.14 (6.58)		20.97 (5.95)	

Table 3 shows the correlation between learning approaches and preferred types of assessments. Those with deeper learning approaches preferred practical exams (standardized or real-patient assessments) and those with more surface approaches preferred multiple-choice questions (Table 3).

**Table 3.** Correlation between Biggs study approach (surface or deep) and methods of evaluation preferred by students

	Biggs Deep	Biggs Surface
SP	0.114**	-0.032
Real patient	0.136**	-0.079*
Student SP	-0.012	0.029
MCQ	-0.169	0.093*
Open	-0.039	0.008
Open and MCQ	-0.018	-0.060
Individual work	-0.018	0.013
Group work	-0.020	0.001

**P < 0.01; *P < 0.05. SP = simulated patient; MCQ = multiple-choice questions.

## DISCUSSION

Medical students’ exhausting routine, as found during this study, has been described extensively in the scientific literature. It includes long hours of study, little sleep, much content absorbed passively, surface learning and a tendency towards greater study loads on days preceding tests.^13^^,^^14^^,^^16^

Nevertheless, our findings attempt not only to describe the study routine, but also to try to understand how surface or deeper learning might influence students. It was found that students with a surface approach had poorer quality of sleep and more frequently chose to study just before the exam; they used materials and summaries originating from classes, cheated more frequently and exhibited greater preference for multiple-choice questions. On the other hand, students with a deep approach chose to read books and preferred practical exams with patients, whether real or simulated. From this data, we observed that the type of approach is highly linked to the students’ study routine and health.

In relation to the quality of sleep, a study on university students in all disciplines showed that their quality of sleep was poorer during testing periods, with fewer hours of sleep and increased reporting of symptoms compatible with insomnia.^13^ Another study, specifically on medical students, showed that students with poorer study outcomes tended to present more sleep-related problems during testing periods.^17^ Those findings were also observed in our study. Students with a surface approach presented poorer quality and fewer hours of sleep before tests than did students with a deep approach. They also used more psychostimulants. This can be explained by their limited strategy of basic memorization of the subject matter close to the test day, without integrating prior knowledge and with probably a worse academic performance that students who used a deep approach. This is related to and corroborated by data found in the literature.^17^

Another point to be highlighted is the practice of cheating. The prevalence of cheating found in our study (46%) was lower than what was reported in a study in India (74%)^18^ but was higher than in Saudi Arabia (29%),^19^ Ethiopia (19.8%)^20^ and the United States (2%).^21^ We found that cheating was greater among students with a more surface learning approach. Those students would have a superficial motive for studying, which was simply pass the subject. Cheating is one way to more easily obtain a passing grade.^2^ Our findings were also in accordance with those of another study in which it was found that students who used books as study resources (deep approach) had a lower rate of dishonesty in academic settings, which included cheating less.^22^ Regarding preferred assessment methods, students with deep learning tended to prefer practical methods of assessment based more on ability and attitude, by means of using real or simulated patients, while those with a more superficial profile tended to prefer multiple-choice tests, which are related to more cognitive and more immediate knowledge.^23^

The above results provide further support for Bigg’s learning approaches theory,^4^ in which students with deep approaches tend to study comprehensively in order to obtain the entire picture as observed in their study resources (books instead of summaries), while students with surface approaches tend to avoid failure at school by doing the minimum needed (cheating, for example) and rely on memory instead of comprehension (choosing multiple-choice assessments and studying late on the night before an exam, as shown by their poor quality of sleep).

These associations could serve to underwrite future interventions regarding changes to the type of learning (from surface to deep) that could influence students’ study routines positively, since such changes would lead to students being less stressed and spending less time memorizing cognitive content in the days preceding assessments. In fact, the use or nonuse of surface or deep strategies is closely related to how certain information is passed on to students. It is influenced by diverse factors, such as professors’ attitudes and enthusiasm, the type of subject studied, methods used for studying and students’ enthusiasm.^1^^,^^3^ Therefore, some curricular changes could be envisaged, such as changing to a student-centered method instead of a teacher-centered method, providing more interaction between students, enhancing contact with patients in the early years of medical training, using active educational strategies (team-based learning, problem-based learning, flipped classroom, games and case-based learning, among others) and developing different types of assessments (portfolio, objective structured clinical examination and Mini-Clinical Evaluation Exercise for Trainees, among others). These could potentially shift students’ learning approaches and also enhance the learning process, which might indirectly impact health outcomes and performance.

McManus et al.^10^ showed that approaches towards knowledge acquisition are dynamic processes. They are influenced not only by ways of studying but also by the means used to evaluate the content. In this context, the study resources used by students may say more about their strategies for assimilating content than might their results from assessments. Wilson and Fowler^1^ compared two groups of students who took a course of the same content and duration, but in which one used a conventional learning and assessment model and the other used an active model. The group that took the course with the active learning model was found to have a higher level of strategies and deeper motives in approaching content.

Educators need to be attentive to the type of learning strategy that their students use, given that the surface approach may be associated with negative consequences for the students who use this approach. Learning approaches can be modified through methods that are centered more on students and on interaction, and through using fewer cognitive assessments.

The present study had some limitations that need to be borne in mind. Although the questionnaire was applied to students in different year groups during the same period of the academic year, the test schedules for each semester are different. Moreover, the stress levels relating to individual students’ extracurricular activities were not evaluated. These factors may have influenced students when filling out the questionnaires. Another detail to be taken into consideration is that individuals with prior sleep disturbances or susceptibility to addiction to psychostimulants may present distinct results within the study population. Considering that learning methods, along with the other variables analyzed, are dynamic processes, there is a need for a longitudinal study. It is also worthwhile pointing out that our results refer only to one Brazilian medical school and, therefore, need to be corroborated in other institutions. Furthermore, this was a quantitative study with application of questionnaires. Use of qualitative methods and semi-structured interviews could help in further understanding our results in future studies. Finally, some factors investigated in the present study (e.g. study patterns/resources and cheating) do not have specific and gold standard instruments in the literature. Therefore, we decided to use questions that we created or adapted from previous studies. This was in line with previous studies published in high-impact journals that have used self-created adapted instruments to assess cheating^19^^,^^20^^,^^21^ and study patterns/resources.^16^^,^^24^^,^

## CONCLUSIONS

The type of learning approach used (surface or deep) is associated with study patterns, preference for type of assessments, practices of cheating and the quality of sleep among medical students. Educators need to be attentive to the type of learning that their students use and need to think about measures that will have a positive impact on the interactivity between teaching methods and assessments, and on students’ quality of learning.
